# Higher serum uric acid level is inversely associated with renal function assessed by cystatin C in a Japanese general population without chronic kidney disease: the KOBE study

**DOI:** 10.1186/s12882-019-1291-4

**Published:** 2019-04-02

**Authors:** Sachimi Kubo, Yoko Nishida, Yoshimi Kubota, Aya Higashiyama, Daisuke Sugiyama, Takumi Hirata, Naomi Miyamatsu, Ayumi Tanabe, Aya Hirata, Yukako Tatsumi, Aya Kadota, Kazuyo Kuwabara, Tomofumi Nishikawa, Yoshihiro Miyamoto, Tomonori Okamura

**Affiliations:** 1 Cohort Study Team, Center for Cluster Development and Coordination, Foundation for Biomedical Research and Innovation at Kobe, 2-2 Minatojima Minamimachi, Chuo-ku, Hyogo 650-0047 Japan; 2 Department of Environmental and Preventive Medicine, Hyogo College of Medicine, 1-1 Mukogawa-cho, Nishinomiya, Hyogo 663-8501 Japan; 3 Department of Preventive Cardiology, National Cerebral and Cardiovascular Center, 5-7-1 Fujishiro-dai, Suita, Osaka 565-8565 Japan; 4 Department of Preventive Medicine and Public Health, Keio University School of Medicine, 35 Shinanomachi, Shinjuku-ku, Tokyo, 160-8582 Japan; 5 Department of Preventive Medicine and Epidemiology, Tohoku Medical Megabank Organization, Tohoku University, 2-1 Seiryo-machi, Aoba-ku, Sendai, Miyagi 980-8573 Japan; 6 Department of Clinical Nursing, Shiga University of Medical Science, Seta Tsukinowa-cho, Otsu, Shiga 520-2192 Japan; 7 Department of Hygiene and Public Health, Teikyo University School of Medicine, 2-11-1 Kaga, Itabashi-ku, Tokyo, 173-8605 Japan; 8 Center for Epidemiologic Research in Asia, Shiga University of Medical Science, Seta Tsukinowa-cho, Otsu, Shiga 520-2192 Japan; 9 Faculty of Health Science, Kyoto Koka Women’s University, 38 Kadonocho, Nishikyogoku, Ukyo-ku, Kyoto 615-0822 Japan

**Keywords:** Chronic kidney disease, Serum uric acid, Cystatin C, A community-based study

## Abstract

**Background:**

Although several epidemiological studies have suggested that high serum uric acid (SUA) levels are related to a decline in kidney function, only a few studies have investigated using cystatin C to calculate estimated glomerular filtration rate (eGFR). We aimed to clarify the relationship between SUA levels and kidney function assessed by cystatin C in a Japanese general community population without chronic kidney disease (CKD).

**Methods:**

We conducted a community-based cross-sectional study that included 1086 healthy participants, aged 40–74 years, without CKD and not undergoing treatment of hyperuricemia, who had participated in the baseline survey of the Kobe Orthopedic and Biomedical Epidemiological (KOBE) study. The preconditions for participation in this study were no past histories of cardiovascular disease or cancer, and not undergoing treatment for diabetes, hypertension, or dyslipidemia. We classified the participants into quartiles stratified by sex according to their SUA level and then examined the relationship with eGFR. The odds ratios for having a low eGFR, defined as the lowest quartile of eGFR (i.e., ≤78.4 mL/min/1. 73m^2^) was estimated according to SUA quartiles (men, Q1 ≤ 5.0, Q2 5.1–5.9, Q3 6.0–6.6, and Q4 ≥ 6.7; women, Q1 ≤ 3.8, Q2 3.9–4.3, Q3 4.4–4.9, and Q4 ≥ 5.0 mg/dL) after adjustment for age, body mass index, systolic blood pressure, HbA1c, high and low density lipoprotein cholesterol, and smoking and drinking habits. The adjusted mean of each quartile was also calculated.

**Results:**

Multivariable-adjusted means of eGFR showed a graded decrease in higher SUA quartiles (men, Q1 90.5, Q2 88.0, Q3 83.5, and Q4 82.0; women, Q1 95.7, Q2 91.3, Q3 89.2, and Q4 86.7). In addition, the multivariable-adjusted odds ratios for having a lower eGFR (95% confidence interval) for each SUA quartile compared with Q1 was Q2 2.29 (0.98, 5.35), Q3 4.94 (2.04, 11.97), and Q4 8.01 (3.20, 20.04) for men, and was Q2 2.20 (1.12, 4.32), Q3 2.68 (1.39, 5.20), and Q4 4.96 (2.62, 9.41) for women.

**Conclusions:**

There was a graded inverse relationship between mild elevations in SUA levels and eGFR assessed by cystatin C in an apparently healthy Japanese population without CKD. This association was similar in both men and women.

## Background

Several epidemiological studies have reported that hyperuricemia is associated closely with renal dysfunction [1–8]. A 7-year follow-up study of Japanese subjects showed that serum uric acid (SUA) levels and increased levels of serum creatinine were significantly related, and that hyperuricemia (≥6.0 mg/dL) was associated with progression of end stage renal disease in women. In addition, maintenance of a normal SUA level was important for maintaining normal renal function [1]. Another follow-up study of Japanese individuals without chronic kidney disease (CKD) reported that the rate of CKD onset in the group with high SUA levels at baseline was greater than that in the group with a lower SUA level, and that the rate of decline in renal function per year was rapid [2]. However, there is insufficient evidence to determine whether or not gender-based SUA levels are associated with a mild decline in renal function before the onset of CKD. The level of estimated glomerular filtration rate (eGFR) assessed by cystatin C is not influenced by either muscle mass, nutrition, or physical activity status and is therefore considered to be a suitable method for evaluating mild renal dysfunction [9].

This cross-sectional study examined the association between SUA level and renal function stratified according to gender in the non-CKD general population who had not been treated for cardiovascular diseases or cancer.

## Methods

### Study participants

We used the data collected in the baseline survey of the Kobe Orthopedic and Biomedical Epidemiological (KOBE) study. The KOBE study has been ongoing since 2010 and is a population-based cohort study of risk factors for worsening of quality of life or cardiovascular risk factors in Kobe City, an urban area in Japan. The KOBE study has been described in detail elsewhere [10–13].

A total of 1117 subjects (341 men and 776 women), ranging in age from 40 to 74 years participated in the baseline survey between July 2010 and December 2011. None of the participants had a past history of cardiovascular disease or cancer, and none were receiving medications for diabetes, hypertension, or dyslipidemia. We excluded 16 subjects with CKD (< 60 mL/min/1. 73m^2^) and 11 subjects who were taking medication for hyperuricemia at baseline. Participants with missing data (*n* = 4) on potential confounding factors were also excluded, leaving a total of 1086 participants (321 men and 765 women) in the data analysis.

### Data collection

The study participants were asked to complete a self-reported questionnaire about lifestyle-related factors, such as history of medication, smoking (current smoker or not) and drinking habits (current drinker or not). The responses were confirmed directly by trained researchers. Daily salt intake was evaluated by estimating the 24-h urinary sodium chloride and potassium excretion using a spot urine, and was calculated by the equation developed by Tanaka et al. [14]. The body mass index (BMI) was calculated as body weight (kg) divided by height squared (m^2^). Blood pressure was measured twice in each participant using an automatic sphygmomanometer (BP-103i II; Nihon Colin, Tokyo, Japan) after a 5-min rest, and the mean value for each participant recorded. Blood samples were collected after a 10-h fast from all participants and then tested in a commissioned clinical laboratory center (SRL Inc., Tokyo, Japan). SUA was measured using the uricase-peroxidase method [15], hemoglobin A1c (HbA1c) level by the latex coagulation method, and serum total cholesterol (TC), high-density lipoprotein cholesterol (HDL-C) and triglyceride (TG) levels by enzymatic methods. Low-density lipoprotein cholesterol (LDL-C) was calculated using Friedewald’s formula [16]. Serum cystatin C was measured using the colloidal gold technique, and eGFR (mL/min/1. 73m^2^) calculated by the following equation developed by the Japanese Society of Nephrology: eGFR_cys_ = 104 × serum cystatin C^− 1.019^ × 0.996^age^ (× 0.929 if female) – 8 [17].

### Statistical analysis

SUA levels were classified into quartiles by sex, and a sex-specific analysis of SUA quartiles then performed. The basic characteristics were presented as means (standard deviation [SD]) for continuous variables, or as percentages for categorical variables. Because the distribution of TG levels was skewed, the data were logarithmically transformed and expressed as median (interquartile range). eGFR classified by quartiles of SUA was also compared using an analysis of covariance after adjustment for age, BMI, systolic blood pressure, HbA1c, HDL-C, LDL-C, and smoking and drinking habits. Bonferroni adjusted tests for significance were used to compare the groups. The participants were also categorized into two groups as follows: high eGFR group (2nd to 4th quartile, > 78.4 mL/min/1. 73m^2^) and low eGFR group (1st quartile; ≤78.4 mL/min/1. 73m^2^). Logistic regression models were used to calculate the odds ratios (ORs) for a low eGFR in participants with higher SUA levels (2nd to 4th quartile of SUA) compared with those in the lowest quartile of SUA. The models were also adjusted for age, BMI, systolic blood pressure, HbA1c, HDL-C, LDL-C, and smoking and drinking habits. All data were analyzed using IBM SPSS Statistics 22.0.

## Results

Tables 1 and 2 shows the sex-specific characteristics of the study subjects stratified by quartiles of SUA levels. The data were expressed as mean (SD). Mean age was 60.8 (8.9) years in men and 57.9 (8.7) years in women, while mean SUA level was 5.9 (1.2) mg/dL in men and 4.4 (0.9) mg/dL in women. The prevalence of hyperuricemia (SUA level > 7.0 mg/dL) was 15.3% in men and 0.4% in women, while mean eGFR was 86.0 (13.7) mL/min/1. 73m^2^ in men and 90.8 (15.3) mL/min/1. 73m^2^ in women. In subjects with higher SUA levels, mean BMI, systolic blood pressure (SBP) and diastolic blood pressure (DBP), median TG and the prevalence of current alcohol drinkers were higher and mean eGFR was lower in both men and women. Mean LDL-C was also higher in women [mean (SD) LDL-C for the lowest and the highest quartile of SUA levels were 118 (28) mg/dL and 122 (25) mg/dL for men and 129 (27) mg/dL and 139 (28) mg/dL for women.], while mean estimated salt intake was not different between men and women with higher SUA levels.

**Table 1:** Characteristics of the male participants grouped according to serum uric acid levels

	Serum uric acid quartile				*P* for trend
	1st (low)	2nd	3rd	4th (high)	
Men (*n* = 321)					
Number of participants	78	84	78	81	
Serum uric acid (mg/dL)	≤5.0	5.1–5.9	6.0–6.6	≥6.7	
eGFR (mL/min/1. 73m^2^), mean (SD)	88.7 (13.9)	88.3 (13.7)	84.4 (13.0)	82.5 (13.6)	0.001
Age (years), mean (SD)	62.7 (7.9)	60.2 (9.1)	59.9 (9.0)	60.3 (9.4)	0.103
BMI (kg/m^2^), mean (SD)	21.7 (2.4)	22.4 (2.4)	22.8 (2.5)	24.0 (2.7)	< 0.001
SBP (mmHg), mean (SD)	120 (18)	121 (17)	123 (16)	127 (17)	0.007
DBP (mmHg), mean (SD)	75 (9)	76 (10)	79 (10)	81 (11)	< 0.001
HbA1c (%), mean (SD)	5.5 (0.5)	5.7 (0.8)	5.5 (0.5)	5.5 (0.4)	0.273
HDL-C (mg/dL), mean (SD)	63 (14)	61 (14)	60 (15)	60 (14)	0.137
LDL-C (mg/dL), mean (SD)	118 (28)	125 (27)	131 (26)	122 (25)	0.180
TG (mg/dL), median (IQR)	68 (49, 92)	78 (58,107)	98 (64, 120)	107 (74, 157)	< 0.001
Current smoker, n (%)	8 (10)	14 (17)	5 (6)	6 (7)	0.201
Current alcohol drinker, n (%)	53 (68)	61 (73)	62 (79)	71 (88)	0.002
Current alcohol intake, ethanol (g/day)^a^, median (IQR)	20 (6, 20)	14 (10, 36)	20 (9, 42)	23 (12, 49)	0.144
Estimated salt intake, g/day (SD)	8.9 (1.9)	9.0 (1.9)	8.7 (2.0)	9.0 (1.9)	0.987

*eGFR* Estimated glomerular filtration rate, *BMI* Body mass index, *SBP* Systolic blood pressure, *DBP*, Diastolic blood pressure, *HDL-C* High-density lipoprotein cholesterol, *LDL-C* Low-density lipoprotein cholesterol, *TG* triglyceride, *IQR* interquartile range. ^a^Current drinker only

**Table 2:** Characteristics of the female participants grouped according to serum uric acid levels

	Serum uric acid quartile				*P* for trend
	1st (low)	2nd	3rd	4th (high)	
Women (*n* = 765)					
Number of participants	204	172	195	194	
Serum uric acid (mg/dL)	≤3.8	3.9–4.3	4.4–4.9	≥5.0	
eGFR (mL/min/1. 73m^2^), mean (SD)	98.1 (16.0)	91.0 (14.2)	89.5 (14.1)	84.1 (13.5)	< 0.001
Age (years), mean (SD)	56.3 (9.2)	58.1 (8.6)	57.5 (8.7)	59.7 (7.9)	< 0.001
BMI (kg/m^2^), mean (SD)	20.0 (2.1)	21.0 (2.5)	20.8 (2.6)	22.1 (3.1)	< 0.001
SBP (mmHg), mean (SD)	109 (15)	113 (16)	114 (17)	117 (18)	< 0.001
DBP (mmHg), mean (SD)	66 (9)	69 (10)	69 (11)	72 (11)	< 0.001
HbA1c (%), mean (SD)	5.6 (0.6)	5.5 (0.4)	5.5 (0.3)	5.6 (0.4)	0.865
HDL-C (mg/dL), mean (SD)	72 (15)	72 (15)	71 (15)	71 (17)	0.279
LDL-C (mg/dL), mean (SD)	129 (27)	136 (29)	133 (30)	139 (28)	0.005
TG (mg/dL), median (IQR)	63 (49, 89)	70 (58, 105)	68 (64, 120)	82 (74, 154)	< 0.001
Postmenopause, n (%)	136 (67)	140 (81)	148 (76)	170 (88)	< 0.001
Current smoker, n (%)	6 (3)	3 (2)	4 (2)	2 (1)	0.213
Current alcohol drinker, n (%)	62 (30)	58 (34)	85 (44)	75 (39)	0.023
Current alcohol intake, ethanol (g/day)^a^, median (IQR)	5 (3, 13)	7 (2, 14)	7 (3, 13)	11 (3.4, 17)	0.559
Estimated salt intake, g/day (SD)	8.2 (1.8)	8.5 (1.8)	8.1 (1.7)	8.2 (1.9)	0.655

*eGFR* Estimated glomerular filtration rate, *BMI* Body mass index, *SBP* Systolic blood pressure, *DBP* Diastolic blood pressure, *HDL-C* High-density lipoprotein cholesterol, *LDL-C* Low-density lipoprotein cholesterol, *TG* Triglyceride, *IQR* Interquartile range. ^a^Current drinker only

**Fig. 1:**
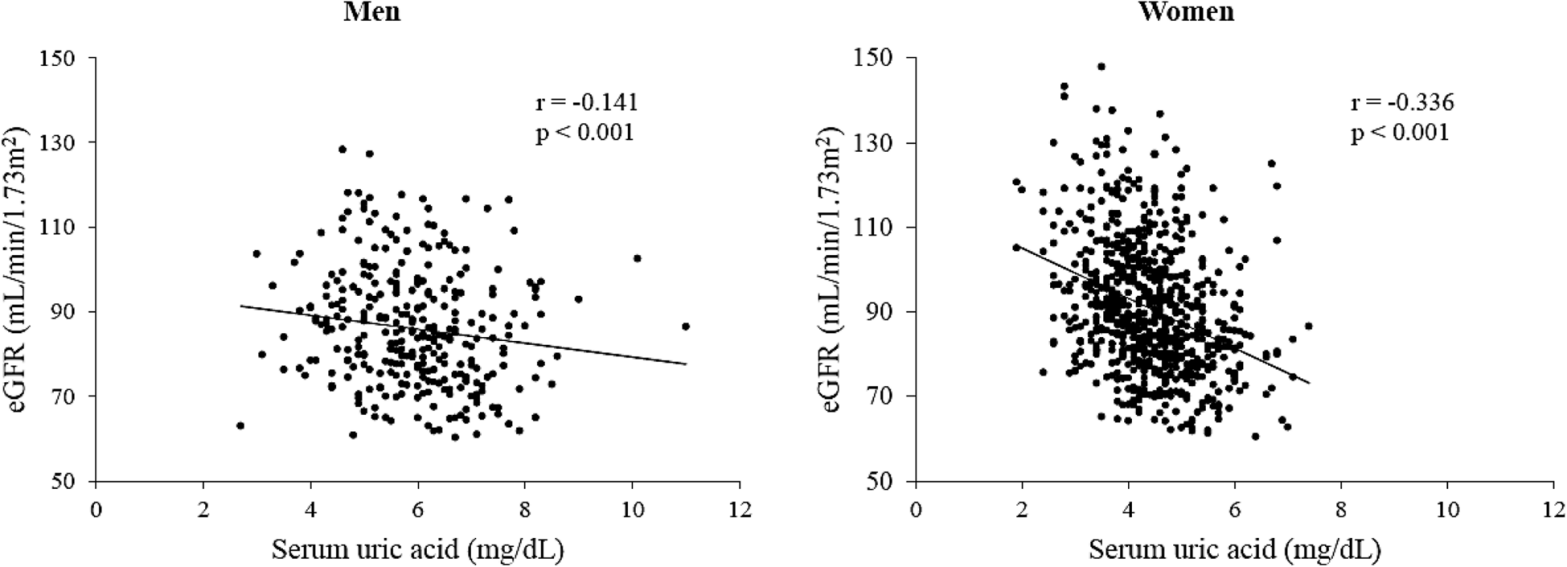
Relationship between SUA level and eGFR, grouped according to gender

Figure 1 shows a sex-specific scatter-graph of SUA and eGFR. SUA levels showed an inverse correlation with eGFR in both men and women (men, *r* = − 0.141, *p* <  0.001; women, *r* = − 0.336, *p* <  0.001).

Age- and multivariable-adjusted means of eGFR grouped according to quartiles of SUA levels are shown in Table 3. Multivariable-adjusted means of eGFR also showed a graded decrease in higher SUA quartiles (men, Q1 90.5, Q2 88.0, Q3 83.5, Q4 82.0; women, Q1 95.7, Q2 91.3, Q3 89.2, Q4 86.7).

**Table 3:** Age- and multivariable-adjusted means of eGFR according to serum uric acid values by gender at baseline

	Serum uric acid quartile			
	1st (low)	2nd	3rd	4th (high)
Men (*n* = 321)				
Serum uric acid (mg/dL)	≤5.0	5.1–5.9	6.0–6.6	≥6.7
Number of participants	78	84	78	81
Age-adjusted eGFR (mL/min/1. 73m^2^)	90.5	87.8	83.6	82.1
Multivariable-adjusted eGFR (mL/min/1. 73m^2^)	90.5	88.0	83.5	82.0
Women (*n* = 765)				
Serum uric acid (mg/dL)	≤3.8	3.9–4.3	4.4–4.9	≥5.0
Number of participants	204	172	195	194
Age-adjusted eGFR (mL/min/1. 73m^2^)	96.3	91.2	89.3	86.1
Multivariable-adjusted eGFR (mL/min/1. 73m^2^)	95.7	91.3	89.2	86.7

Multivariable adjustment; Adjusted by age, BMI, SBP, HbA1c, HDL-C, LDL-C, smoking habit and drinking habit

*eGFR* Estimated glomerular filtration rate, *BMI* Body mass index, *SBP* Systolic blood pressure, *HDL-C* high-density lipoprotein cholesterol, *LDL-C* Low-density lipoprotein cholesterol

**Fig. 2:**
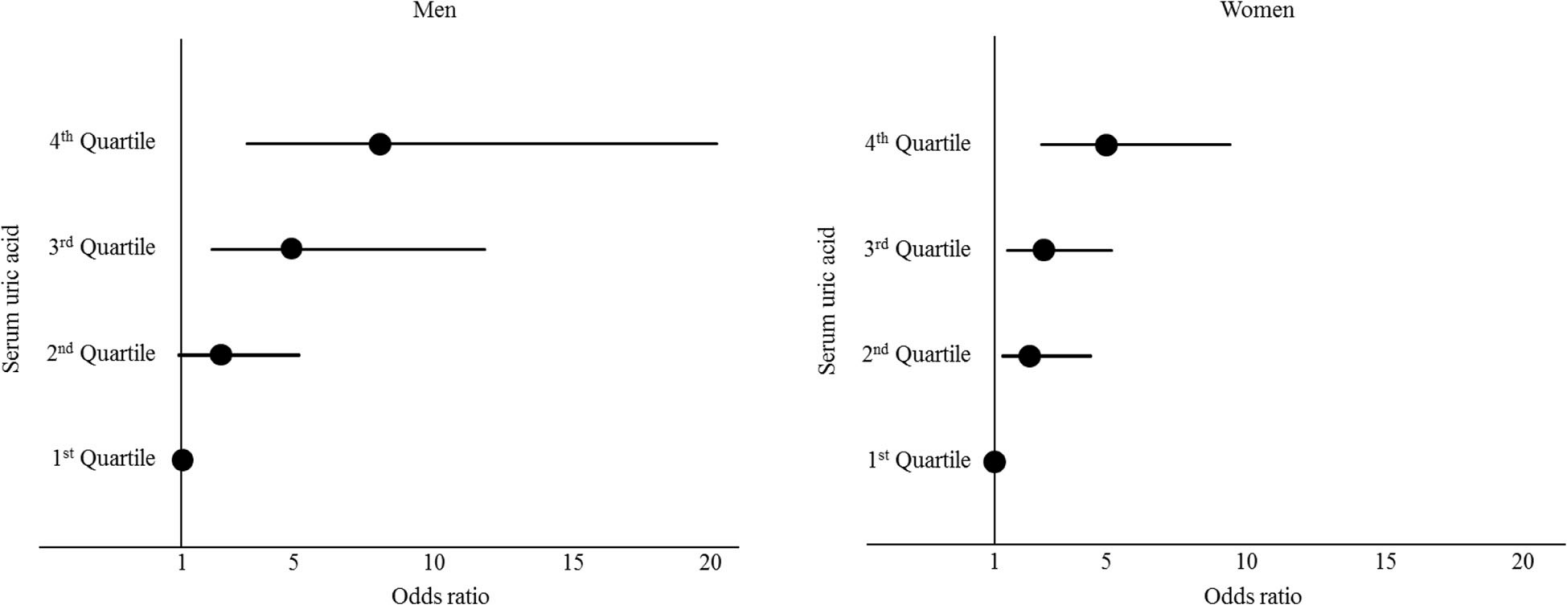
Multivariable-adjusted odds ratios of the lowest eGFR groups, grouped according to serum uric acid levels. Multivariable adjustment; Adjusted by age, BMI, SBP, HbA1c, HDL-C, LDL-C, and smoking and drinking habits. eGFR, estimated glomerular filtration rate; CI, confidence interval; BMI, body mass index; SBP, systolic blood pressure; HDL-C, high-density lipoprotein cholesterol; LDL-C, low-density lipoprotein cholesterol. Serum uric acid quartiles: men, Q1 ≤ 5.0, Q2 5.1–5.9, Q3 6.0–6.6, and Q4 ≥ 6.7; women, Q1 ≤ 3.8, Q2 3.9–4.3, Q3 4.4–4.9, and Q4 ≥ 5.0 mg/dL

The multivariable-adjusted ORs for having a low eGFR (eGFR ≤78.4 mL/min/1. 73m^2^) grouped according to SUA levels are shown in Fig. 2. For both men and women, the ORs increased significantly and gradually in all SUA quartiles, with the exception of the 2nd quartile in men. The age-adjusted ORs (95%CI) of the lowest eGFR group for the highest SUA quartile were 5.30 (2.42, 11.61) in men and 5.83 (3.16, 10.74) in women [men, Q2 1.85 (0.84, 4.06), Q3 3.67 (1.67, 8.06), Q4 5.30 (2.42, 11.61); women, Q2 2.36 (1.22, 4.58), Q3 2.85 (1.50, 5.40), Q4 5.83 (3.16, 10.74)]. This association became more evident after multivariable-adjustment in men [men, Q2:2.29 (0.98, 5.35), Q3 4.94 (2.04, 11.97), Q4 8.01 (3.20, 20.04); women, Q2 2.20 (1.12, 4.32), Q3 2.68 (1.39, 5.20), Q4 4.96 (2.62, 9.41)]. The multivariable-adjusted ORs of the lowest eGFR group for a 1 mg/dL increment in SUA were 1.45 (1.14, 1.86) in men and 1.84 (1.47, 2.29) in women.

## Discussion

The present study showed that higher SUA levels were associated inversely with eGFR levels assessed by cystatin C in the general community population without CKD or a past history of cardiovascular disease or cancer, and not undergoing treatment for diabetes, hypertension, dyslipidemia or hyperuricemia.

In our study subjects, 49 men (15.3%) and 3 women (0.4%) had the criteria for hyperuricemia. And the results of women were in a population that included few subjects clinically diagnosed hyperuricemia.

The results of the present study showed a graded inverse association between SUA levels and eGFR assessed by cystatin C in both men and women. A previous study of healthy Japanese subjects (19 men and 29 women) who were scheduled to provide a kidney for transplantation (mean age 54.6 ± 13.4 years) reported that SUA level showed a significant U-shaped association with GFR measured by inulin clearance [18]. However, the sample sizes of this earlier study and the present study (48 vs. 1086) were markedly different, and also this other study [18] performed a sex-combined analysis.

The Hisayama Study which was a 5-year follow-up study of the 2424 Japanese subjects in the general population (1042 men and 1382 women aged over 40 years) free from CKD at baseline reported that the multivariable-adjusted ORs (95%CI) for developing CKD (eGFR < 60 mL/min/1. 73m^2^ assessed by serum creatinine [19] or urine albumin-creatinine ratio ≥ 30 mg/g) were significantly increased with higher SUA levels within the normal range [SUA ≤4.0 mg/dL: reference, SUA 4.1–4.9 mg/dL, 1.22 (0.87, 1.72); SUA 5.0–5.8 mg/dL,1.57 (1.11, 2.22); SUA ≥5.9 mg/dL, 2.18 (1.49, 3.18)] [2]. The Atherosclerosis Risk in Communities Study and the Cardiovascular Health study in 13,338 subjects in the US population (5789 men and 7549 women, aged over 45 years) with an average 8.5-year follow-up period and eGFR calculated using serum creatinine by the Modification on Diet in Renal Disease Study equation [20], showed that baseline SUA level was associated with a significantly increased risk for developing a decline in kidney function (decrease in eGFR of ≥15 mL/min/1. 73m^2^ and final eGFR < 60 mL/min/1. 73m^2^; OR per 1 mg/dL increase in baseline SUA (95%CI). 1.07 (1.01, 1.15)] [3]. Although these studies used sex combined analyses and eGFR assessed by serum creatinine calculated by different equations from those used in our study, the results obtained were consistent with our findings.

There was a marked gender difference in SUA level, which was higher in men than women. One reason is higher prevalence of current alcohol drinker in men than women. Higher BMI in men than women also attributed to high SUA in men although mean BMI level in the present study is not high. Another reason is uricosuric effect of estrogen. Of 765 women in the present study, 171 were premenopausal women, of which SUA levels (4.1 mg/dL) was lower than those of postmenopausal women (4.5 mg/dL) and men (5.9 mg/dL), although it was difficult to perform subgroup analysis by status of menopause. We performed sex-specific analysis because SUA level distribution between men and women was quite different. On the other hand, when we changed the SUA cut-off points of men in Fig. 2 to the same ones of women, we could observe consistent results irrespective of gender [The multivariable-adjusted ORs (95%CI) of the lowest eGFR group for the highest SUA group, of which cut-off point was the highest quartile in women, were 4.54 (0.97, 21.31) in men and 4.96 (2.62, 9.41) in women], however approximately 80% (257/321) of men classified in this category. In other words, prevalence of possible high-risk individuals due to high SUA in men was much higher than that in women.

Although there is no gender difference in SUA saturation concentration, regardless of gender or age, SUA levels > 7.0 mg/dL are defined as hyperuricemia in Japan [21]. However, a previous Japanese study reported that an increased SUA level was a significant and independent risk factor for cardiovascular mortality in women with SUA levels > 6.0 mg/dL mg/dL and there was no relation in men [22]. The Evidence for Cardiovascular Prevention from Observational Cohorts in Japan Study that had a median follow-up of 10 years showed almost similar results; i.e., cardiovascular mortality in women was increased in women with SUA levels > 5.1 mg/dL, although in this study, increased risk of cardiovascular disease was observed in men with SUA levels > 6.7 mg/dL [23]. Taken together, this evidence may suggest that women need to pay more attention to the potential risks of hyperuricemia from lower SUA levels than men [24]. However, we should pay more attention not only relative risk of high SUA but also on absolute risk such as real mortality or prevalence in men. When we classified men according to statistical distribution of SUA, such as quartile or quintile, they had potentially high absolute risk of cardiovascular disease or impaired renal function or even in the lower quartiles or quintile.

Several observational studies have already reported the relationship between SUA level and CKD and onset of CVD [25–30]. However, there are no clinical research reports on this association. For this reason, a long-term observation in clinical trials is necessary to clarify that the development of renal dysfunction caused by high normal SUA levels progresses to the onset of CKD and subsequently to onset of CVD.

Although we could not elucidate the mechanism of the results found in our study, a previous study reported a mechanism whereby SUA may cause a decline in renal function, in which SUA induces production of active oxygen species following uptake of SUA into the cell. Uric acid activates mitogen-activated protein kinase and nuclear transcription factors, consequently, increases cyclooxygenase-2 production, which causes inflammation in the vascular endothelium, proliferation of vascular smooth muscle, and intrarenal vascular lesion through transporters such as urate transporter 1 [31]; uric acid also activates renin-angiotensin system, which causes systemic and glomerular hypertension associated with renal dysfunction [32]. For example, after withdrawal of allopurinol in patients with chronic kidney disease and mild hyperuricemia, increase in blood pressure and worsening of serum creatinine were only observed in patients without being under medication by pharmacological blockers of the renin-angiotensin system [33]. Renal arteriopathy occurred in hyperuricemic rats; these were prevented by administration of allopurinol or benziodorone [34].

Our study had several strengths. First, our study subjects were selected from an apparently healthy population who did not have a history of CKD, cardiovascular disease or cancer, and also had no medication history for hypertension, diabetes, dyslipidemia and hyperuricemia. Accordingly, we were able to study an almost natural association between SUA and renal function. Second, we used cystatin C to evaluate eGFR which is more suitable for evaluating renal function in healthy individuals than creatinine. Third, we examined the relationship between SUA levels which were not categorized clinically as hyperuricemia and minor reductions in kidney function prior to CKD.

This study also had a few limitations. First, the study used a cross-sectional dataset in the analyses. However, we are continuing to carry out the KOBE study and therefore it will be possible to analyze a longitudinal dataset in the future. Second, we set the classification for lower CKD value as quartile 1 of the eGFR distribution (eGFR ≤78.4 mL/min/1. 73m^2^). The reason why we used this classification was that the proportion of subjects in the G2 group of CKD (eGFR 60–89 mL/min/1. 73m^2^) in this study was large (66% of men and 54% of women). However, because the eGFR range of G2 was relatively wide, the range of Q1 in the eGFR 78.4 mL/min/1. 73m^2^ or lower group in the study subjects was approximately the same as the median of the G2 classification. We therefore speculate that it may be a meaningful indicator of renal dysfunction in the preclinical stage of CKD.

## Conclusions

The present study showed that there was an inverse relationship between a mild elevation in SUA level and eGFR assessed by cystatin C in a healthy Japanese general population without CKD. A study on changes in eGFR using follow-up data of the KOBE study is required in the future.

## Abbreviations

BMI: Body mass index
CI: Confidence interval
CKD: Chronic kidney disease
DBP: Diastolic blood pressure
eGFR: estimated glomerular filtration rate
HbA1c: Hemoglobin A1c
HDL-C: High-density lipoprotein cholesterol
IQR: Interquartile range
KOBE: Kobe Orthopedic and Biomedical Epidemiological
LDL-C: Low-density lipoprotein cholesterol
ORs: Odds ratios
SBP: Systolic blood pressure
SD: Standard deviation
SUA: Serum uric acid
TC: Total cholesterol
TG: Triglyceride
